# Leadership Learning Through Short‐term Study Abroad Courses

**DOI:** 10.1002/yd.70036

**Published:** 2026-02-16

**Authors:** Evan Witt, Tara Coste

**Affiliations:** ^1^ Leadership Minor University of Minnesota Minneapolis Minnesota USA; ^2^ Leadership & Organizational Studies University of Southern Maine Portland Maine USA

## Abstract

When exploring the landscape of leadership learning in study abroad, it is critical to understand the leadership learning that takes place through short‐term study abroad courses. This article will provide a definition of short‐term study abroad and short‐term study abroad leadership courses through an overview of relevant literature. It will further examine leadership study's connection to the development of global leadership competencies. Implications for universities, students, and leadership educators will be provided.

## 1 Introduction

Why does higher education emphasize students studying abroad? After all, it is expensive, and, when done right, involves a significant time investment for both faculty and students. The opportunity for study abroad courses to provide an environment that is rich with opportunities for deep leadership learning makes it worth the effort. To make this important enrichment tool available to students from a variety of income levels and household types, in traditional and non‐traditional environments, we particularly recommend an investment in short‐term study abroad. Short‐term study abroad is any study abroad experience that takes place for less than eight weeks (IIE 2024). In the 2022–2023 academic year, this accounted for 61% of study abroad programs (IIE 2024).

Short‐term study abroad is often delivered in a faculty‐led (as opposed to an independent) format. Faculty‐led study abroad is defined as a “credit‐granting, college‐level study abroad program where faculty accompany students from their universities as teachers and trip leaders” (Keese and O'Brien 2011, p. 5). A growing trend in leadership education is the emergence of short‐term leadership study abroad courses. *Short‐term study abroad leadership development courses* are academic courses with curriculum centered on the study and practice of leadership within a global environment (Montgomery and Arensdorf 2012).

Throughout this article we will focus on leadership learning opportunities for short‐term study abroad courses more broadly as well as focusing on short‐term study abroad leadership courses specifically. After a review of the related literature, we will leverage our own experiences to identify applications, strengths, challenges, and implications for educators. This article will provide an important overview of the overall landscape of global leadership learning through short‐term study abroad courses.

### 1.1 Author Positionalities

Our analysis of leadership learning through short‐term study abroad courses will be informed by our own identities and lived experiences. Evan identifies as a white straight cisgender male, regularly navigating the privileges associated with those identities, especially in the leadership education space. He has lived internationally in several global contexts, in addition to regularly working with students and colleagues from different countries. He teaches a short‐term study abroad leadership course in Ireland and Northern Ireland with a focus on social change. Tara moved out of the United States at eight weeks old, spending her formative years in Southeast Asia. Based on this foundation, her work has centered on untangling the interpersonal dynamics that influence leadership effectiveness in post‐colonial environments. These lines of investigation are now focused on how ancestral belief systems drive modern behavior and *The Montagu Project*, a multi‐decade global leadership lab in South Africa.

## 2 Relevant Literature

### 2.1 Short‐Term Study Abroad

Study abroad is a high‐impact pedagogical practice and has demonstrated student outcomes including intercultural learning, world‐mindedness, academic benefits, and personal growth (Douglas and Jones‐Rikkers 2001; Engle and Engle 2004; Kuh 2008; Miller‐Perrin and Thompson 2010). Although semester and year‐long study abroad programs have been the most common model historically, over 60% of current programs now use a short‐term study abroad model (IIE 2024). The trend toward short‐term study abroad programs is important and provides an opportunity to maximize leadership learning across an expanded audience of study abroad students.

A short‐term study abroad model can alleviate student barriers to studying abroad, particularly for historically minoritized students, including cost, family concerns, institutional factors, and lack of relevance (Brux and Fry 2010). However, Strange and Gibson (2017) found program lengths equal to or greater than two and a half weeks produce similar transformative learning results, while shorter programs produce reduced results, comparatively. Therefore, programs shorter than two‐and‐a‐half weeks risk having less impact on student learning. Students in short‐term study abroad programs that grant sufficient time for immersive experiences are associated with positive outcomes, including awareness of other cultures, appreciation of other cultures’ impact, and personal identity awareness; however, they may not result in outcomes related to complex situations, respect for cultural perspectives, and a sense of responsibility to others (Gaia 2015). Thus, carefully curating educational experience is critical.

### 2.2 Short‐Term Study Abroad Leadership Courses

Of particular interest for our discussion is leadership learning. Academic leadership courses are one of the most popular methods for facilitating student leadership development (Mitchell and Daugherty 2019; Rosch and Jenkins 2020). While leadership development is not a stated outcome of every study abroad program, there is a growing number of leadership‐focused study abroad programs which provide robust opportunities for leadership learning (Niehaus 2018). In fact, a number of U.S. universities have institutionalized international leadership experiences that take place through study abroad (e.g., the School of Leadership Studies at Gonzaga University, the McDonough Center for Leadership & Business at Marietta College, the Jepson School of Leadership Studies at University of Richmond, and the School of Leadership and Education Sciences at University of San Diego; Armstrong 2020). However, it is not enough to simply offer these programs. Evaluating leadership learning is also important (Armstrong 2020; Beatty and Manning‐Ouellette 2022; Montgomery and Arensdorf 2012).

Numerous examples capture the impact of leadership learning in short‐term study abroad courses. For example, one Midwestern U.S. institution provided key course objectives used when creating their study abroad leadership courses (Montgomery and Arensdorf 2012). A more recent study used the Intercultural Effectiveness Scale as a pretest‐posttest for a cohort of approximately 20 students over a five‐year period from 2013–2017 (Armstrong 2020). A short‐term study abroad leadership course used assessment that demonstrated that participants grew in the areas of leadership efficacy and capacity (Beatty and Manning‐Ouellette 2022). Furthermore, a qualitative study of students’ understanding of global leadership through short‐term leadership study abroad courses demonstrated how global experiences were important in understanding global leadership and the importance of well‐crafted curriculum in enhancing students' understanding of global leadership (Witt 2024). Collectively, these studies demonstrate multiple approaches to the complex leadership learning outcomes in short‐term study abroad courses.

### 2.3 Global Competencies

But what specifically are these global competencies leadership educators seek to develop in students? How might they be best prepared to meet the demands of a highly interconnected global economy? Deardorff's (2006) foundational study found that 80 to 100 percent of top intercultural scholars identified the elements below, in Table 1, as key to intercultural competence (p. 249):

**TABLE 1:** Deardorff's elements of intercultural competence.

Ability to communicate effectively and appropriately in intercultural situations based on one's intercultural knowledge, skills, and attitudes. Ability to shift frame of reference appropriately and adapt behavior to cultural context; adaptability, expandability, and flexibility of one's frame of reference/filter. Ability to identify behaviors guided by culture and engage in new behaviors in other cultures even when behaviors are unfamiliar given a person's own socialization. Behaving appropriately and effectively in intercultural situations based on one's knowledge, skills, and motivation. Ability to achieve one's goals to some degree through constructive interaction in an intercultural context Good interpersonal skills exercised interculturally; the sending and receiving of messages that are accurate and appropriate. Transformational process toward enlightened global citizenship that involves intercultural adroitness, intercultural awareness, and intercultural sensitivity.

*Note*: Adapted from, (Deardorff 2006).

The study further found that 80 to 100 percent of both scholars and administrators identified the following as specific components of intercultural competence (pp. 249–250), as seen in Table 2:

**TABLE 2:** Specific components of intercultural competence.

Understanding others’ worldviews Ethnorelative view. Cultural self‐awareness and capacity for self‐assessment. Skills to listen and observe. Adaptability and adjustment to new cultural environment. Flexibility. Ability to adapt to varying intercultural communication and learning styles. Tolerating and engaging ambiguity. Deep knowledge and understanding of culture (one's own and others’). Respect for other cultures. Understanding of role and impact of culture and the impact of situational, social, and historical contexts involved.	Cross‐cultural empathy. Learning through interaction. General openness toward intercultural learning and to people from other cultures. Understanding the value of cultural diversity. Cognitive flexibility—ability to switch frames from etic to emic and back again. Mindfulness. Sociolinguistic competence (awareness of relation between language and meaning in societal context) Withholding judgment. Culture‐specific knowledge and understanding host culture's traditions. Curiosity and discovery. Skills to analyze, interpret, and relate.

*Note*: Adapted from, (Deardorff 2006).

Two decades later, research continues to support these findings, and study abroad is an important strategy to build global competencies (Fisher et al. 2022). It challenges students’ perspectives by inspiring them to reach beyond their individual cultural boundaries. It develops competence and knowledge of global concerns. It influences attitudes, communication, and learning by exposing them to rapidly changing environments that require flexibility and adaptability. It “fosters personal growth, intercultural development, and career attainment” (Fisher et al. 2022, pp. 421–422).

### 2.4 Identity Exploration and Reformation

Around the time of Deardorff, another scholar developed a model that was equally illuminating. In 2004, Hunter published the model of global competence. After extensive work with a diversity of stakeholders, Hunter identified components of global competence that build internal or external states of readiness (p. 115), seen in Table 3.

**TABLE 3:** Hunter's (2004) model of global competence.

Attitudes (internal readiness)	Knowledge (external readiness)	Skills/Experiences (external readiness)
1. Recognition that one's own worldview is not universal. 2. Willingness to step outside of one's own culture and experience life as “the other”. 3. Willingness to take risks in pursuit of cross‐cultural learning and personal development. 4. Openness to new experiences, including those that could be emotionally challenging. 5. Coping with different cultures and attitudes. 6. A non‐judgmental reaction to cultural difference. 7. Celebrating diversity.	1. Understanding one's own cultural norms & expectations. 2. Understanding cultural norms & expectations of others. 3. Knowledge of world history. 4. Knowledge of current world events. 5. Understanding the concept of globalization.	1. Ability to identify cultural differences. 2. Ability to live outside one's own culture. 3. Ability to collaborate across cultures. 4. Successful participation on academic or work projects with people from other cultures. 5. Ability to assess intercultural performance in social or professional settings. 6. Effective participation in socially and professional settings globally.

*Note*: Adapted from, (Hunter 2004).

*Global learning* asks students to critically engage with global environments, to become informed, flexible, and mindful of diversity across all differences so that they may understand how their behavior affects people far and wide and that they share responsibility for addressing the world's most important concerns (Hoff and Medina 2022). Study abroad programs designed to build community, encourage exploration, and require reflection create safe spaces to explore the self, and self in relation to others (Simopoulou et al. 2022). Short‐term study abroad enables students to move past their comfort zones and gain a better understanding of their identities from multiple perspectives (Murray et al. 2015). Intercultural competence is critical for those who want to function effectively in local and international contexts as they navigate an increasingly interconnected world (Ji 2020).

### 2.5 Study Abroad as Experiential Pedagogy

Chase's (2021) in‐depth examination of the experiential learning element of study abroad contributes further important information. He argued developing intercultural competence within study abroad requires “increasing cultural self‐awareness, deepening understanding of the experiences, values perceptions, and behaviors of people from diverse communities, and the capacity to shift cultural perspectives and adapt behaviors to create understanding across cultural differences” (p. 39). He further noted how study abroad and experiential learning opportunities combined lead to unique forms of student learning in civic awareness, intercultural competence, and global knowledge, among others. Of course, reality is constructed through perception, and deeper perception results from more complex intercultural experiences.

In mission statements across the U.S., colleges and universities have prioritized intercultural competency so that students may succeed in the modern world. Yet institutions of higher education must help students to develop the necessary competencies to be prepared to adapt to and thrive in this rapidly changing global economy. Sadly, higher education literature is rife with the assumption that simply exposing students to other ways of being in the world will result in global awareness and world‐mindedness (Volpe‐White 2024; Witt 2024). However, it is only experiential study abroad pedagogy that deliberately takes its students beyond international travel or tourism that can facilitate the development of global citizenship and the attainment of strong global competencies (Chase 2021). The pedagogy of experiential study abroad is grounded by the concept that intentional design, cultural immersion, and structured opportunities for reflection and analysis will lift lived experience to a place in which those experiences can connect to broader academic, cultural, and personal outcomes (Kiely 2004; Woolf 2006).

### 2.6 Assessment of Outcomes

As more students take part in study abroad programs, stakeholders (students, faculty, and administrators) are asking if the investment in study abroad will truly help develop the skills students need in today's global community. High‐quality assessment of study abroad programs examine the effectiveness of learning outcome attainment. Thus, an outcomes‐based approach to evaluating study abroad programs mandate an assessment of the learning experience(s) (Maharaja, 2018).

Unfortunately, there are few examples of instruments developed to apply across a broad cross‐section of industries and cultural contexts (Strong et al. 2020). Cartwright et al. 2021, pp. 97‐99) provided a detailed exploration of one such example, the Intercultural Effectiveness Survey (IES), which may be used for pre‐ and post‐program assessment. The dimensions it measures are found in Table 4.

**TABLE 4:** Dimensions & subfactors of the intercultural effectiveness survey.

Continuous Learning Dimension	Interpersonal Engagement Dimension	Hardiness Dimension
Measures the degree to which participants engage the world by continually seeking to understand and learn about the activities, behavior, and events that occur around them.	Measures participants’ interest in other cultures and the importance of developing relationships with people from other cultures.	Measures participants’ ability to effectively manage their thoughts and emotions in intercultural situations, along with their ability to be open‐minded and nonjudgmental about ideas and behaviors that are new to them.
Self‐Awareness subfactor: The degree to which participants are aware of their personal values, strengths, weaknesses, interpersonal style, and behavioral tendencies, as well as the impact of these things on other people.	World orientation subfactor: The degree to which participants are interested in, and seek to actively learn about, other cultures and the people that live in them.	Open‐Mindedness subfactor: The degree to which participants withhold judgments about situations and people that are new or unfamiliar to them.
Exploration Subfactor: The extent to which participants are open to and pursue an understanding of ideas, values, norms, situations, and behaviors that are different from their own.	Relationship interest subfactor: The extent to which participants are likely to initiate and maintain positive relationships with people from other cultures.	Emotional resilience subfactor: Participants’ level of emotional strength and their ability to cope with challenging emotional experiences.

*Note*: Adapted from, Cartwright et al. (2021).

Post‐secondary institutions in the U.S. benefit from robust assessments to justify their study abroad programs in a climate with increasing costs and limited resources. The tool above is just one of the approaches colleges and universities can implement to respond to the call for the internationalization of higher education and to provide students with opportunity to acquire the competencies necessary to succeed in a diverse global environment (Majarajah, 2018).

### 2.7 Access to Study Abroad

Success in higher education is often measured by enhanced student recruitment, retention, and graduation rates. High‐impact programming is particularly important for the retention of at‐risk student populations. Education abroad is clearly a high‐impact experience, but first generation, minority, and transfer students are underrepresented in those who participate. Students taking loans and students who need help from others to pay for college also participate in study abroad at a much lower rate than their peers (McGrew et al. 2021). Expenses are a major factor in access to study abroad, especially for students at public universities.

In fact, lack of financial resources and insufficient financial aid limit study abroad opportunities for students from historically minoritized backgrounds (Brux and Fry 2010). Universities must address this concern. Study abroad courses allow these students to develop closer relationships with faculty members and other students so that they may become more integrated (socially, academically) with university culture (Murray et al. 2015). In sum, it is important for all students to be provided the opportunity “to examine different cultures and their perspectives on society, systems, politics, and relationships through experiential, hands‐on opportunities” (Bletscher and Hellmann 2022, p. 157).

### 2.8 Institutional Support

Education abroad is a practice that can be traced back to the Middle Ages, but there has been a notable increase in its popularity over recent decades (Hoffa 2007). Not coincidentally, the number of publications on international education has doubled every decade since the 1970s (Varela and Gatlin‐Watts 2014). While the number of participants in study abroad programming was the original measure of success, the literature review presented above demonstrates that this work has become much more complex.

To examine what exactly was under investigation, Varela and Gatlin‐Watts (2014) undertook a meta‐analysis of study abroad publications. They found 16% of publications in *The Interdisciplinary Journal of Study Abroad* and the *Journal of Studies in International Education* focused on exploring drivers of students’ participation. Three factors—personality traits, prior travel experience, and gender—were regularly found to be antecedent to study abroad. A second batch of studies (about 18%) explored the institutional factors that support study abroad. These studies highlighted the role that universities’ consortia, scholarships, and study abroad offices play, illuminating the logistical and financial elements underpinning a program's success.

Institutions of higher education are not universally supporting international education effectively. Universities should engage in practices that maximize meaningful interactions among diverse students. Unfortunately, research suggests many universities fail to support international education and intercultural interaction on their campuses at a time when it is more important than ever (Bletscher and Hellmann 2022). As an example, in a culture of higher education where missions regularly extoll internationalization and the importance of intercultural experiences, faculty are not usually rewarded for the amount of work these programs place on top of their other responsibilities (Niehaus 2019). If an institution's infrastructure, policies, and procedures do not communicate the importance of international education, it is unlikely to be widely embraced. When global mobility is on the rise, higher education must continue to shift and change to meet the needs of a global workforce, and incentivizing both faculty and students is essential (Savishinsky 2012; Sutton and Obst 2011).

### 2.9 Identifying Gaps in Literature

As previously described, research in the fields of study abroad and student leadership is extensive, yet the intersection of research across the two fields is more limited, and a specific focus on global leadership competencies is scarcer still. A research gap for student leadership is especially true in short‐term study abroad programs which have become the dominant model of study abroad. Thus, there are a number of gaps in the literature that this article aims to address.

Short‐term study abroad provides a rich environment for global leadership learning, yet literature rarely measures this specific outcome. Researchers need both quantitative and qualitative studies to more fully demonstrate this outcome. Scholars should collect additional data using validated leadership instruments. Particularly valuable research would capture students’ global leadership competencies before and after these experiences.

While the debate will continue about the optimal length for short‐term study abroad courses, there is an opportunity to articulate specific leadership outcomes regardless of course length. The establishment of research driven best practices for short‐term leadership study abroad courses could help enhance leadership outcomes in a short time frame. For example, it has been established that carefully crafted reflective course assignments before, during, and after the experience will help students identify their individual behavioral tendencies, strengths, weaknesses, and values and how these attributes may impact their interactions with others (Niehaus et al. 2012).

Finally, leadership learning in short‐term study abroad is deeply contextual based on the location of the study abroad experience as well as the individual identities of the students participating in the programs. There is a need for research that explores leadership learning based on different student identities such as race, gender, and sexuality, among others. Individual identities will intersect with the cultural context of leadership learning to create distinctly different types of leadership learning outcomes.

For these gaps to be addressed, a practice‐based scholarship approach is needed to link research with the realities of short‐term study abroad course delivery. Examples include applied assessments that capture leadership learning in diverse study abroad contexts or mixed method longitudinal research designs that capture the full scope of leadership learning from pre‐departure through long‐term reflections. Practice‐based scholarship can help translate the complexities of leadership learning in short‐term study abroad courses into strategies that drive student outcomes across varied program lengths, locations, and student populations.

## 3 Applications

It is important to acknowledge that short‐term study abroad is and will likely continue to be the most popular model for education abroad experiences. Thus, focusing on leadership learning through this model is crucial. The short‐term model provides more access for students to engage in education abroad due to the lower financial and time commitments compared to semester and year‐long study abroad programs.

While the short‐term study abroad model is well established, short‐term leadership study abroad courses are still an emerging phenomenon. Short‐term leadership study abroad courses provide an opportunity for students to observe and interpret their leadership learning in specific global contexts. Institutionalizing this practice can help contribute to institutional goals related to preparing students to engage as future global leaders.

One such project is The Montagu Project (Coste and Neethling 2025). The Montagu Project came about due to leadership students wanting an immersive experience in disadvantaged communities in South Africa. In response, a global learning lab was located in rural South Africa which houses a learning center that facilitates leadership education in Montagu and neighboring communities. Undergraduate and graduate students from a variety of majors travel to South Africa regularly in short‐term study abroad courses that center on learning from and with leaders in this context. During the rest of the year, students maintain relationships with the South African program participants, create further programming to share cross‐continents, and write grants and develop other funding sources to support this work. As the outcomes of the project materialized, the initiative became a signature program of the Department of Leadership and Organizational Studies and well‐supported by the University of Southern Maine.

## 4 Strengths and Challenges

There are a number of strengths and challenges to explore related to leadership learning in short‐term study abroad. Short‐term study abroad experiences are more accessible to students due to their lower cost and time commitment. This can help diversify the students who study abroad, which is currently mostly White and women (IIE 2024). Another strength lies in the ability of students to apply their leadership skills through experiential learning in these new global contexts. Finally, the outcomes related to study abroad as a whole directly align with the outcomes of many leadership models including personal awareness, awareness of others, and the ability to impact change.

The challenges of short‐term study abroad center on the duration of the experience. Experiences that are less than eight weeks may provide a less immersive experience and opportunity for leadership learning (Dwyer 2004). It can equally be challenging to assess leadership learning due to the intensity of the experiences and ongoing reflective activity. Longitudinal studies could better capture aspects of the ongoing leadership learning that may occur well after students complete a short‐term study abroad experience.

### 4.1 Implications

It is important to dispel the myth that by simply engaging in a global experience students will automatically develop leadership capacities. Leadership learning in short‐term study abroad must be carefully facilitated and curated through intentional curriculum, experiences, and reflection. Upon reviewing the literature and supported by our own experiences, we have provided a number of key implications for educators and institutions looking to enhance leadership learning through short‐term study abroad.

Fundamentally, it is critical that these initiatives are addressed in three primary areas, using institutional, pedagogical, and student‐focused lenses simultaneously. First, institutions must make funding available for the creation and maintenance of short‐term study abroad programs. Cost serves as a significant barrier in the development of and participation in short‐term study abroad programs. Parallel to institutionalizing faculty support is a need for subsidizing student costs so that a broader swath of the student body can participate in these transformative leadership learning experiences. As institutions put the proper support mechanisms in place, faculty must focus on deliberate program design that strategically develops the pre‐departure, in‐country, and re‐entry elements of the learning process. Finally, a plan of integrated assessment practices should evaluate student outcomes, particularly in the areas of identity exploration and leadership efficacy.

This article provides a summary of what is currently understood about the opportunity for leadership learning in short‐term study abroad programs. With this knowledge, leadership educators will be better equipped to make the case at their institutions for the creation and support of short‐term study abroad courses. Future research agendas that can continue to advance this knowledge base should look across the totality of student identities, emphasize leadership learning as a specific course outcome in short‐term study abroad programs, and highlight best practices to more effectively facilitate leadership learning in globally focused curricula.
